# Collagens from Marine Organisms towards Biomedical Applications

**DOI:** 10.3390/md20030170

**Published:** 2022-02-25

**Authors:** Azizur Rahman, Tiago H. Silva

**Affiliations:** 1 Centre for Climate Change Research, University of Toronto, ONRamp, Toronto, ON M5G 1L5, Canada; 2 A.R. Environmental Solutions, ICUBE-University of Toronto, Mississauga, ON L5L 1C6, Canada; 3 3B’s Research Group, I3Bs–Research Institute on Biomaterials, Biodegradables and Biomimetics, University of Minho, Headquarters of the European Institute of Excellence on Tissue Engineering and Regenerative Medicine, AvePark-Parque de Ciência e Tecnologia, Zona Industrial da Gandra, 4805-017 Barco, Guimarães, Portugal; 4 ICVS/3B´s-PT Government Associate Laboratory, 4806-909 Guimarães, Braga, Portugal

Collagen is the main fibrous structural protein in the extracellular matrix and connective tissue of animals. It is a primary building block of bones, tendons, skin, hair, nails, cartilage, and all joints in the body. It is also considered a "glue" that holds the body together. In this regard, it receives great attention in healthcare and wellbeing, both as a functional ingredient in different formulations and as a component of several products, such as medical devices and pharmaceutical systems. The production of collagen begins to slow down, and cell structures start losing their strength as we get older, and supplementing with collagen is being explored as a vital way to help our body revive and stay youthful. Indeed, a wide range of products comprising collagen (or collagen hydrolysates, in most cases) can be found, such as lotions, creams, face masks and even nutraceuticals. On the other hand, collagen-based biomedical materials have also been developed, with important and clinically effective materials gaining wide acceptance.

However, collagen extraction from land animal sources is complex, time-consuming and expensive. Moreover, there are some concerns over adverse inflammatory and immunologic response and the prevalence of various diseases among land animals which causes health complications, resulting in ethical and regulatory constraints, pushing industry—and scientists—to look for alternatives. Hence, marine sources have started to be researched and have been found to be quite convenient and safe for obtaining collagen. The main advantages being claimed over the land animal sources are (1) a high content of collagen; (2) environmentally friendly; (3) the presence of biological contaminants and toxins being almost negligible; (4) low inflammatory response; (5) greater absorption due to low molecular weight; (6) less significant religious and ethical constraints; (7) minor regulatory and quality control problems.

Marine resources for the production of collagen include both invertebrates and vertebrates, such as sponges, coralline red algae, sea urchins, octopi, squids, jellyfish, cuttlefish, starfish, sea anemones, and prawns; in addition to the different species of fish, the latter has the advantage that much biomass being used for collagen extraction is a by-product of fish processing for food, such as fish skins, scales, etc., thus offering both economic and environmental benefits. Moreover, several applications for marine collagens have been studied and proposed, promising a great contribution to marine biotechnology products and medical applications in the short term. Aware of the significant scientific relevance of marine collagens and the pivotal role they can assume in human health, we edited this Special Issue comprising a series of original studies and reviews on the biological sources of these proteins, including production methodologies and on their promising applications in medical and related fields.

In particular, the collagens that can be found in the different marine invertebrates and ways to extract them have been overviewed in a review paper [1], followed by another review focusing particularly on the marine sponges [2], an ancestral group of animals widely studied for their biological role played in marine ecosystems, and additionally for being an untapped source of bioactive compounds. Herein, a special focus can be found on three sponge species: *Axinella cannabina*, *Suberites carnosus* and *Chondrosia reniformis*. The former two were the subject of a study by Tziveleka et al. [3], where insoluble collagen, intercellular collagen, and spongin-like collagen were extracted (Figure 1) and characterized envisaging biotechnological application. 

The latter sponge species, *Chondrosia reniformis*, is well known for its richness in collagen, and different methodologies have been proposed for its extraction, enabling the production of hydrogel-like materials with interesting rheological behavior and sticky features [4]. Moreover, collagen membranes derived from sponges were assessed for their water-binding capacity, antioxidant activity, and cytocompatibility envisaging skincare application [5]. Considering the biomedical relevance promised by this sponge collagen, efforts have been made on the aquaculture of *Chondrosia reniformis* [6], aiming to establish a sustainable route providing the biomass needed for collagen production. Still, in invertebrates, other phyla have been also researched, namely Cnidaria, in which jellyfish have been particularly explored considering the biomass availability resulting from blooms. In this regard, Bernhardt et al. [7] studied the use of *Rhopilema esculentum* jellyfish collagen for the development of biphasic scaffolds (interestingly, in combination with another marine collagen, from salmon skin) for osteochondral tissue engineering. Other cnidarians also inspiring scientists are corals, with Benayahu et al. [8] studying the unique collagen fibers identified in *Sarcophyton* soft corals and further combining them with alginate to produce biocomposite hydrogels for biomedical application.

Despite the advancements achieved with marine invertebrate collagens and the intriguing features exhibited by some of these organisms, such as the dynamic collagenous tissues observed in echinoderms, fish (vertebrates) are, by far, the most explored group of marine animals for collagen production. Different species have been studied, researching several parts of the animal, namely skin, scales, bones, swim bladders and fins. In this Special Issue, studies can be found addressing collagen from giant croaker (*Nibea japonica*) [9], codfish (*Gadus morhua*) [10], Nile tilapia (*Oreochromis niloticus*) [11,12], pufferfish (*Takifugu flavidus*) [13], small-spotted catshark (*Scyliorhinus canicula*) [14], common smooth-hound (*Mustelus mustelus*) [15] and blue shark (*Prionace glauca*) [16]. These species are, in general, already known for the production of collagen, with the herein-reported studies exploring advances in the extraction methodologies and the evaluation of applicability of these collagens, mostly regarding human health. In this perspective, the optimization of extraction conditions, such as acid and enzyme concentrations, biomass-to-solution ratio, temperature or time of reaction, has been pursued [9], including by the application of specific statistical tools, such as response surface methodology (RSM) [14]. In addition, new purification techniques have been also proposed, such as electrodialysis that promises economic and environmental advantages over traditional dialysis [13]. Moreover, thorough characterization of collagen was implemented by different authors to understand the main properties of the isolated materials, namely the molecular weight, by assessing the electrophoretic profile, the denaturation temperature, extract purity, and effect on cell behavior, in which a positive effect was observed over the cell adhesion or proliferation of fibroblasts [10], endothelial cells [11], osteoblasts [11,16] and mouse bone marrow–mesenchymal stem cells [16].

The field of biomaterials and biomedical applications is expanding at an unprecedented pace, trying to face the challenges of a growing population and longer life expectancy, and collagen-based devices are playing a relevant role. In this perspective, this Special Issue includes studies using marine collagen for the development of different types of products, such as wound dressings [12], bioactive films [15], membranes [5] and scaffolds for tissue regeneration [7,8]. With the same rationale, the use of collagen on bone substitutes for facial bone reconstruction has been systematically reviewed by Cicciù et al. [17] and, although not clarifying the advantages of biomaterials vs. autologous bone, the use of marine collagens seems to provide dimensional stability and growth factor carrier versatility to the constructs.

In addition to the use of the integral protein, the use of collagen hydrolysates and peptides is also of great interest for healthcare and wellbeing, as illustrated by the study reported by Ito et al. [18], in which a pilot study in humans has shown the benefits of a supplement comprising fish collagen peptide in the improvement of skin conditions, including elasticity and hydration.

Overall, we hope that this Special Issue elucidates the importance of marine collagens in biomedicine, appearing as an alternative to mammal collagens. It demonstrates the wide biodiversity that can be explored as a raw material for the production of collagen. Additionally, it provides representative examples of biomedical products and devices being proposed for wound healing, tissue regeneration and globally improving human health. This is a growing field, not only among scientists, but also in industry and public policies, and although delivering valuable information, this Special Issue reveals only a small part of the puzzle, hopefully triggering the curiosity of the readers to research further and contribute to the new knowledge being built.

## Figures and Tables

**Figure 1:**
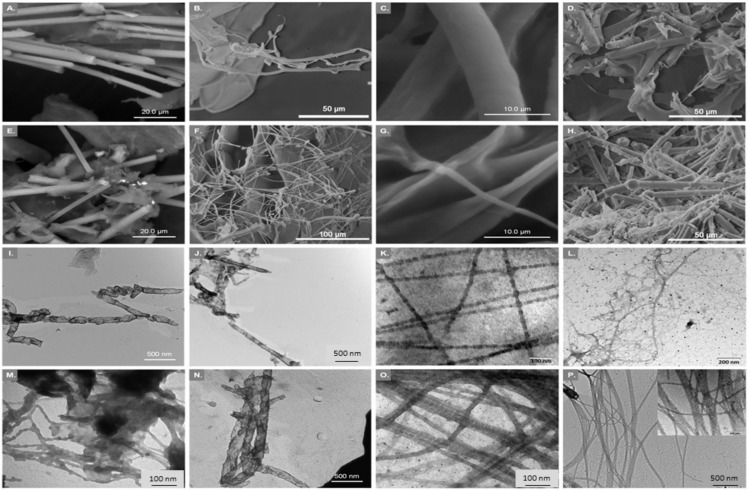
SEM micrographs of insoluble collagen (InSC; **A**,**E**), intercellular collagen (ICC; **B**,**C**,**F**,**G**), and spongin-like collagen (SlC; **D**,**H**) from *Axinella* *cannabina* (row 1) and *Suberites* *carnosus* (row 2), respectively. TEM micrographs of insoluble collagen before (InSC; **I**,**M**) and after (SF-InSC; **J**,**N**) spicule removal, intercellular collagen (ICC; **K**,**O**) and spongin-like collagen (SlC; **L**,**P**) from *Axinella* *cannabina* (row 3) and *Suberites* *carnosus* (row 4), respectively.
